# Metabolic Bone Disease of Prematurity: Risk Factors and Associated Short-Term Outcomes

**DOI:** 10.3390/nu12123786

**Published:** 2020-12-10

**Authors:** Alejandro Avila-Alvarez, Adela Urisarri, Jesús Fuentes-Carballal, Natalia Mandiá, Andrea Sucasas-Alonso, María L. Couce

**Affiliations:** 1 Neonatology Unit, Pediatrics Department, Complexo Hospitalario Universitario de A Coruña, 15006 A Coruña, Spain; jesus.fuentes.carballal@sergas.es (J.F.-C.); andrea.sucasas.alonso@sergas.es (A.S.-A.); 2 INIBIC-Health Research Institute of A Coruña, 15006 A Coruña, Spain; 3 Faculty of Medicine, Universidad de Santiago de Compostela, 15704 Santiago de Compostela, Spain; adela.urisarri.ruiz.de.cortaza@sergas.es; 4 Neonatology Department, University Clinical Hospital of Santiago de Compostela, 15704 Santiago de Compostela, Spain; natalia.mandia.rodriguez@sergas.es; 5 IDIS-Health Research Institute of Santiago de Compostela, 15704 Santiago de Compostela, Spain; 6 CIBERER, Instituto Salud Carlos III, 28029 Madrid, Spain

**Keywords:** metabolic bone disease, prematurity, osteopenia, phosphate, alkaline phosphatase, red blood cell

## Abstract

Despite the importance of early recognition of metabolic bone disease (MBD) of prematurity, there is still significant variability in screening practices across institutions. We conducted an observational study of infants born at ≤32 weeks of gestation with a birth weight of ≤1500 g (n = 218) to identify clinical factors associated with biochemical indicators of MBD. Bone mineral status was assessed by measuring alkaline phosphatase and phosphate levels between weeks 3 and 5 of life. Two comparisons were performed after classifying infants as either MBD (cases) or non-MBD (controls), and as either high or low risk for MBD, as determined based on the results of MBD screening. In total, 27 infants (12.3%) were classified as cases and 96 (44%) as high-risk. Compared with controls, MBD infants had a significantly lower gestational age and birth weight, and a longer duration of parenteral nutrition and hospital stay. Respiratory outcomes were significantly poorer in high- versus low-risk infants. Multivariate logistic regression showed that birth weight was the only independent risk factor for MBD (odds ratio [OR]/100 g, 0.811; confidence interval [CI95%], 0.656–0.992; *p* = 0.045) and that birth weight (OR/100 g, 0.853; CI95%, 0.731–0.991; *p* = 0.039) and red blood cell transfusion (OR, 2.661; CI95%, 1.308–5.467; *p* = 0.007) were independent risk factors for high risk of MBD. Our findings provide evidence of risk factors for MBD that could help clinicians to individualize perinatal management. The association of red blood cell transfusion with MBD is a novel finding that may be related to iron overload and that merits further study.

## 1. Introduction

Despite recent advances in neonatal care, metabolic bone disease (MBD) is still a common complication in preterm newborns who are deprived of the fetal mineral accumulation that normally occurs during the third trimester of gestation [1,2,3].

MBD of prematurity is a complex entity characterized by suboptimal bone matrix mineralization and biochemical alterations of phospho-calcium metabolism. During fetal and early life, mineralization of the bones is influenced by mineral supply (calcium and phosphorus), hormonal factors (parathyroid hormone, estrogens, vitamin D), environmental factors (concomitant diseases, long term parenteral nutrition, immobilization, medications), and genetic predisposition [4]. Although multifactorial in origin, MBD is considered to be primarily the result of reduced mineral accretion during the first weeks of life following preterm birth [5].

MBD is associated not only with rickets and fractures, but also lower peak bone mass, impaired postnatal growth, and poorer respiratory outcomes [1,4,6,7], which in turn have been linked to poorer development later in life and susceptibility to other chronic diseases [4,8,9]. MBD involves more than suboptimal bone mineralization in premature babies, and its prevention is among the highest priorities in modern neonatal care. The basic approach to MBD prevention involves improving calcium and phosphate intake from the first day of life, limiting the use of medications that increase bone resorption or calcium loss, promoting enteral feeding, and early identification of at-risk babies. Routine biochemical screening is recommended in very low birth weight (VLBW) infants in order to individualize subsequent management [10]. This analytical evaluation of bone-related parameters is largely based on the presence of risk factors, but there is significant variation in screening practices across institutions [6,11,12,13,14,15,16]. Moreover, while recent significant advances in the management of VLBW infants (e.g., early aggressive parenteral nutrition, donor breast milk, human milk fortifiers) may have influenced the picture of MBD and increased survival rates, there are limited data on risk factors for MBD that could help clinicians to guide management [17,18,19,20]. The aim of the present study was to identify clinical factors that are associated with biochemical indicators of MBD identified in analytical screening in very preterm infants.

## 2. Materials and Methods

### 2.1. Study Design and Population

A retrospective (1 January 2015, to 31 July 2019) and prospective (1 August 2019, to 31 July 2020), observational multicenter study was conducted at two hospitals in Spain. All infants who were born at ≤32 weeks of gestation with a birth weight of ≤1500 g and were admitted to the neonatal intensive care unit (NICU) in either of the two participating centers were eligible for inclusion. This population was previously reported to have the greatest risk for MBD. Infants with major congenital malformations or chromosomal abnormalities, and those who died or were transferred to another institution before the first biochemical screening for MBD, were excluded. The participating centers were two tertiary referral centers that together attend approximately 8000 deliveries per year. The study was approved by the Local Ethics Committee of each of the participating centers (Santiago code: 2020/265 v2, Coruña code: 2017/360 v3) and written informed consent was obtained from all the participants.

The following variables were recorded at recruitment: demographic data at birth and at screening (gestational age, birth weight, sex); perinatal characteristics (antenatal steroid treatment, preeclampsia, chorioamnionitis); clinical outcomes (necrotizing enterocolitis, patent ductus arteriosus, intraventricular hemorrhage, bronchopulmonary dysplasia (BPD), retinopathy of prematurity, days on mechanical ventilation, days on supplemental oxygen, mortality, hospital stay, growth); administration prior to screening of medications that potentially alter calcium metabolism (postnatal steroids, diuretics, caffeine, red blood cell (RBC) transfusions); nutritional management variables (duration of parenteral nutrition (PN), first day and type of enteral feeding, use of fortifiers, mineral supply); and laboratory analyses for MBD screening (serum alkaline phosphatase (ALP), phosphorus (P), calcium (Ca), magnesium (Mg), 25OH-vitamin D (25-OHD), parathyroid hormone (PTH), and urinary P and Ca).

Only necrotizing enterocolitis ≥ grade 2, as defined by Bell et al., was considered [21]. Patent ductus arteriosus was diagnosed by cardiac ultrasound and managed according to local protocols, and only recorded if medically or surgically treated. Intraventricular hemorrhage was defined and graded according to Volpe [22]. BPD was defined as the need for supplementary oxygen for at least 28 days and classified as moderate or severe depending on oxygen requirements and ventilator support at 36 weeks postmenstrual age [23]. All infants were screened for retinopathy of prematurity, which was graded according to national guidelines [24]. Small for gestational age (SGA) was defined as a birth weight z-score <−1.5. Postnatal growth restriction was defined as a decrease in z-score of >1 between birth and discharge [8]. The rate of weight gain in g/kg/day was calculated using the method described by Patel et al. [25].

Reference intervals for biochemical parameters were as follows [26,27,28]: ALP, 18–80 pg/mL; P, 3.1–5.6 mg/dL; Ca, 8.6–10.3 mg/dL; Mg, 1.5–2.2 mg/dL; 25-OHD levels were considered normal at >20 ng/mL, while levels of 12–20 ng/mL and ≤12 ng/mL were considered indicative of 25-OHD insufficiency and deficiency, respectively. The reference interval for urinary Ca and P was 1–2 mmol/L in both cases [29]. In patients with a PTH determination, hyperparathyroidism was defined as a serum PTH > 88 pg/mL [30].

### 2.2. Nutrition Protocol

The two centers that participated in the study have nutritional management protocols for preterm newborns that are generally in line with current recommendations [31,32,33]: PN from day 0 with an initial protein intake of 2 g/kg/day and an energy intake of approximately 40 kcal/kg/day, with targets of 3.5–4 g/kg of proteins and 100–120 kcal/kg at the end of first week, early initiation of trophic feedings, discontinuation of PN as soon as the newborn can tolerate 100 mL/kg/day of enteral feeding, and fortification of breast milk at the same time. As part of the parenteral nutrition protocol, calcium and phosphorus supplementation began at 2 mmol/kg/day and 1 mmol/kg/day, respectively, and was subsequently adjusted based on levels of ionized calcium, energy, proteins, and fluid intake. All infants began oral vitamin D (400 IU/day) supplementation after discontinuing PN. Oral calcium or phosphorus supplementation was introduced into the nutritional protocol based on the results of the first biochemical screening for MBD. No significant changes to the protocol occurred during the study period, except for the availability of human donor milk in both units from the end of 2016.

### 2.3. MBD Screening

Bone mineral status was determined at the first biochemical screening for MBD, which occurred as standard practice in both centers between 3 and 5 weeks of life. ALP >900 IU/L and serum phosphate levels <5.5 mg/dL (sensitivity of 100% and specificity of 70% for diagnosis of MBD using dual-energy X-ray absorptiometry (DXA)) [34] were used as cutoff values to define MBD in our cohort. Infants that fulfilled only one of these two criteria (ALP >900 IU/L or serum phosphate <5.5 mg/dL) were considered at high risk for MBD. No radiographs were performed to evaluate osteopenia during the study period. In a subset of patients, urinary excretion of calcium, phosphate, and creatinine was determined using spot urine samples taken at the moment of plasma screening [29].

### 2.4. Anthropometric and Analytical Measurements

Weight and length z-scores and percentiles were calculated using the Fenton growth reference [35]. Patients were weighed before their first feed in the morning. Concentrations of P, Ca, Mg, and ALP were determined using standard procedures with the Advia 2400 Analyzer (Siemens Diagnostic Systems, Erlangen, Germany), and 25-OHD and PTH with the Advia Centaur XP Analyzer (Siemens Healthcare Diagnostics, Erlangen, Germany).

### 2.5. Statistical Analysis

Descriptive data are presented as the mean ± standard deviation (IQR) or n (%). For univariate analyses the Student *t*-test, Mann–Whitney U-test, or Yuen test were used for continuous variables and the chi-squared test or Fisher exact test for categorical variables. Cramer’s V was used to measure the strength of association between categorical variables. *p* values < 0.05 were considered statistically significant.

A multivariate logistic regression analysis was performed to assess independent predictors of MBD. All potentially predictive variables that were statistically significant (i.e., *p* < 0.1) in the univariate analysis and those that, although not significant, were clinically relevant, were included in the multivariate analysis. Those with very low incidence and cases with missing values for any of the independent variables were excluded from the analysis. To assess multicollinearity, the variance inflation factor was used. The final multivariate model was constructed using a lasso logistic regression model. *p*-values obtained were adjusted using the Bonferroni correction. Only adjusted *p*-values < 0.05 were considered statistically significant. Analyses were performed using R programming software version 4.0.2 and IBM SPSS version 24.

## 3. Results

### 3.1. Characteristics of the Study Population

**Figure 1:**
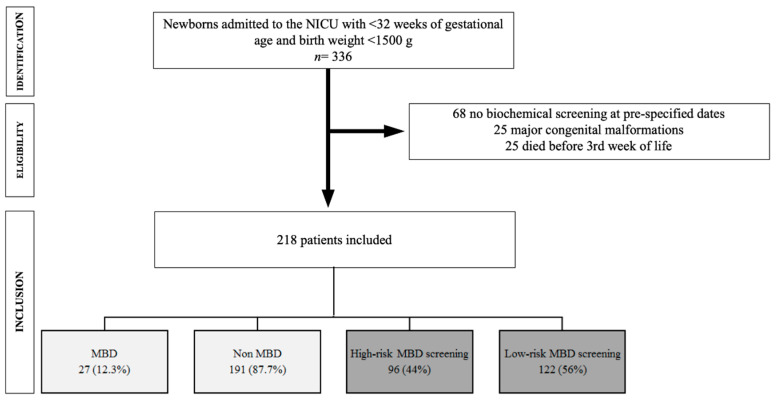
Flow chart depicting recruitment of cohort.

During the study period, a total of 336 patients were born at the two participating centers at ≤32 weeks of gestation with a birth weight of ≤1500 g. After excluding 25 patients with congenital malformations, 25 patients who died before biochemical screening, and 68 patients in which screening was not performed between weeks 3 and 5 of age, a total of 218 infants (125 females) were included in the study (Figure 1). The mean gestational age and birth weight of the study population were 29.9 ± 1.9 weeks and 1207 ± 245 g, respectively. Demographic and baseline characteristics, as well as main outcomes and the results of biochemical screening of participating infants are shown in Table 1. No deaths occurred after inclusion in the study.

Mean age at biochemical screening was 19.7 ± 7.1 days. A total of 27 infants (12.3%) met the previously defined biochemical criteria for MBD and were classified as cases. The remaining 191 patients (87.7%) made up the control group. A total of 96 infants (44%) were classified as high-risk infants and compared with 122 non-high-risk infants (56%).

### 3.2. Univariate Analysis of Study Population

There were no significant differences between cases and controls in terms of sex distribution or incidence of maternal arterial hypertension, chorioamnionitis, SGA, BPD, or enterocolitis. See Table 2 for numerical data and further details.

**Figure 2:**
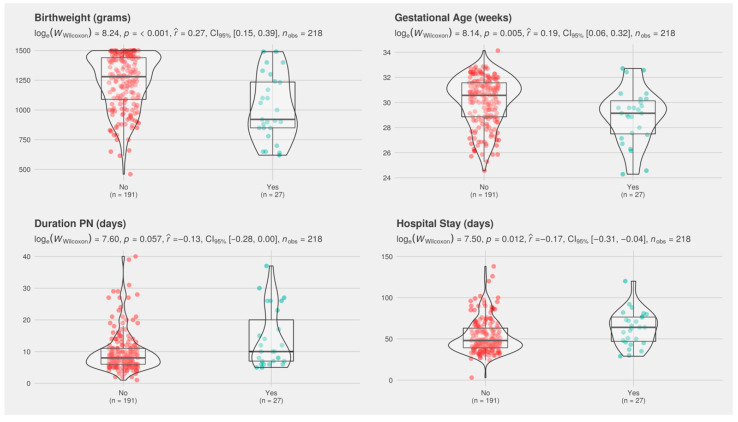
Boxplot merged with a violin plot (a rotated kernel density plot on either side) comparing gestational age, birth weight, hospital stay, and duration of parenteral nutrition (PN) in patients with metabolic bone disease (MBD, cases) versus controls. ‘Yes’ indicates MBD cases and ‘No’ indicates non-MBD patients. In box plots, the boundary of the box closest to zero represents the 25th percentile, the line within the box represents the median, and the boundary farthest from zero represents the 75th percentile. Lines above and below the box represent the 10th and 90th percentiles, respectively. Red and green dots represent the variable in question for cases and controls, respectively. Corresponding mean and interquartile ranges for each variable are provided in Table 2. Violin plots represent kernel density estimation and depict the distribution shape of the data; the probability that infants of the population will take on the given value is higher in wider areas and lower in narrower areas.

Compared with controls, infants with MBD had a significantly lower gestational age (GA) and birth weight, as well as a longer duration of PN and hospital stay (Figure 2).

**Figure 3:**
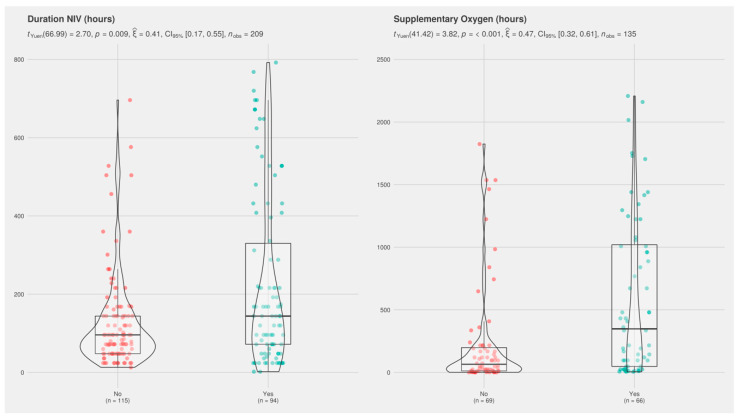
Boxplot merged with a violin plot (a rotated kernel density plot on either side) comparing duration of noninvasive ventilation (NIV) and supplementary oxygen in infants with high versus low risk for metabolic bone disease (MBD). ‘Yes’ indicates high-risk for MBD patients and ‘No’ indicates low-risk patients. In box plots, the boundary of the box closest to zero represents the 25th percentile, the line within the box represents the median, and the boundary farthest from zero represents the 75th percentile. Lines above and below the box represent the 10th and 90th percentiles, respectively. Red and green dots represent the variable in question for cases and controls, respectively. Corresponding mean and interquartile ranges for each variable are provided in Table 2. Violin plots represent kernel density estimation and depict the distribution shape of the data; the probability that infants of the population will take on the given value is higher in wider areas and lower in narrower areas.

Cases also showed a higher incidence of late-onset sepsis and leukomalacia, and more frequently required RBC transfusion. Moreover, high-risk infants showed worse respiratory outcomes (higher rate of surfactant and mechanical ventilation, higher incidence of BPD, and longer duration of non-invasive ventilation and supplementary oxygen) (Figure 3).

In patients in whom urinary excretion of Ca, P, and creatinine was measured, no significant differences were observed between groups except for lower urinary *p* values in patients with MBD (Supplementary Table S1: Urinary excretion of calcium and phosphate in spot urine samples).

### 3.3. Multivariate Analysis

Two multivariate analyses were performed for two dependent variables (MBD and high risk for MBD). Multivariate logistic regression showed that the most important variable associated with the development of MBD was birth weight. When birth weight was included in the logistic regression, most of the factors associated with an increased risk of MBD in the univariate analysis were no longer significant. The results indicate that birth weight was the only independent risk factor for MBD (OR/100 g, 0.811; CI95%, 0.656–0.992; *p* = 0.045) and that birth weight (OR/100 g, 0.853; CI95%, 0.731–0.991; *p* = 0.039) and RBC transfusion (OR, 2.661; CI95%, 1.308–5.467; *p* = 0.007) were independent risk factors for high screening risk for MBD.

## 4. Discussion

Premature babies are at increased risk of developing bone disease due to reduced bone mineral content. In this study, using early biochemical criteria to identify infants at greater risk of MBD, we detected a prevalence of MBD of 12.3%, similar to that previously reported in the literature [2,15,36]. This finding underscores the importance of MBD as a comorbidity in preterm infants. We found no association between MBD and other previously described risk factors, such as postnatal steroid treatment or chorioamnionitis [2,11,18,37]. However, in line with prior reports on the epidemiology of MBD, we found that low birth weight was the most important factor associated with MBD.

The improved survival of preterm infants has coincided with an increase in the number of patients at high risk of MBD. While differences in terminology and diagnostic criteria preclude the identification of the true incidence of MBD [1], available data indicate that even with current nutritional support, MBD remains a significant comorbidity, affecting approximately 15–40% of VLBW infants [2,15,36]. In our series, 12.3% of infants met our previously defined biochemical criteria for MBD between weeks 3 and 5 of life. Moreover, 44% of infants partially fulfilled these criteria and were considered at high-risk of MBD.

Although MBD can self-resolve during early childhood, its potential long-term consequences highlight the importance of current practices to prevent this condition [7]. Despite the crucial importance of early recognition of asymptomatic high-risk infants in order to individualize treatment, as well as screening practices and mineral supplementation, available data on perinatal risk factors for MBD are scarce. In fact, high-risk criteria are based more on clinical experience or individual preferences than on studies specifically designed to address this question [14,16]. Moreover, because neonatal care is changing rapidly and continuously, data published only a few years ago may not fully reflect the landscape of prematurity nowadays [16].

In their 2014 retrospective study, Viswanathan et al. compared a cohort of 71 MBD infants born at <30 weeks of gestation with 159 healthy controls [2]. The MBD group had a significantly lower GA and birth weight than the control group, and significantly greater use of PN, diuretics, and steroids. In 2017, Ukarapong et al. published a retrospective case-control study of 76 infants born at <30 weeks of gestation and evaluated the association of various factors with the likelihood of developing MBD, comparing patients who met their criteria for MBD (n = 40) with those who did not (n = 36). Their results showed that birth weight, duration of PN, cholestasis, medically managed seizures, and prolonged diuretic use were all associated with MBD [37]. Similarly, Chen et al. recently examined risk factors for MBD in an observational cohort study of 16 infants diagnosed with MBD and 32 controls [18]. Using logistic regression analysis, they showed that gestational age <30 weeks and achievement of total enteral nutrition beyond 28 days of age were independent risk factors for MBD [18]. There are several important differences between each of these studies, ours included, particularly relating to the criteria used to define MBD, the sample size, and the variables studied. Despite these differences, many of the factors found to be associated with MBD were common to all studies; infants with MBD were consistently smaller at birth, more premature, and had a longer duration of PN, a longer hospital stay, and greater short-term morbidity [2,18,37].

The decisive influence of birth weight on the incidence of MBD highlights the importance of prenatal mineralization on final bone mineral density. In fact, birth weight has been reported as the clinical variable with the highest positive correlation with bone mineral density in preterm infants as assessed by DXA at discharge [38,39]. Low birth weight can be associated with placental insufficiency, and any condition that impairs placental function and, consequently, nutritional transfer, can lead to increased risk of MBD. These conditions should include those associated with chronic placental damage, such as preeclampsia. However, we observed no significant differences in the incidence of maternal hypertension or SGA between cases and controls. By contrast, mean birth weight z-score was lower in patients with MBD (−0.87 vs. −0.46; *p* = 0.024). Chorioamnionitis, which has been previously associated with reduced bone mass, was not identified as a risk factor in our cohort. However, diagnosis of chorioamnionitis in our cohort was based on medical chart review, not on pathological examination, which is the gold standard, and this may have limited proper assessment of this association.

A novel and interesting finding of the present study is the observed association between RBC transfusion and MBD, which remained significant for high-risk infants in the multivariate analysis. The scarcity of relevant data makes interpretation of this association difficult. RBC transfusion is an important supportive therapy in the management of preterm infants. However, there is a growing body of evidence suggesting that RBC transfusion may also have harmful effects [40]. Unfortunately, MBD is not typically included among the morbidities commonly reported in observational studies characterizing the adverse effects of RBC transfusions in preterm infants or in randomized controlled trials comparing different transfusion thresholds [40,41]. In other conditions at other ages, such as thalassemia in children and adults, iron overload resulting from multiple RBC transfusions is one of the factors implicated in the pathogenesis of osteoporosis observed in these patients [42]. Preterm infants, who receive multiple RBC transfusions, may also be predisposed to iron overload, resulting mainly from accelerated breakdown of transfused RBCs [43,44]. While iron overload, which is implicated in oxidative stress, does not appear to significantly influence liver function [44], iron deposition in the bone may impair osteoid maturation and inhibit mineralization locally, resulting in focal osteomalacia [42]. Sodium citrate provides an alternative explanation for the observed association between RBC transfusion and MBD. Citrate is the anticoagulant of choice used in blood sample collection, and is known to cause a number of electrolytic disturbances, including hypocalcemia [45]. This citrate-related hypocalcemia is usually transitory, and occurs only after massive transfusions in critically ill patients or patients with liver failure. However, we cannot rule out the possibility that citrate toxicity may have also influenced MBD screening in our cohort. Unfortunately, we did not record additional data on transfusion practices (age at transfusion, transfusion thresholds, number of transfusions), iron metabolism (ferritin, transferrin, iron supplementation), or post-transfusion electrolyte changes that could potentially help us elucidate the exact role of RBC transfusions in the pathophysiology of MBD. We attempted to retrieve some data to study this association in our prospective cohort. However, only 11 patients were transfused in this group and none developed MBD. Moreover, although we attempted to adjust for confounders in the multivariate analysis, we cannot rule out the possibility that RBC is simply an indicator of the degree of sickness in this group of babies. Therefore this finding, which merits further study, should be interpreted with caution.

Some drugs frequently used in the NICU have been previously associated with MBD in preterm infants. Examples include hypercalciuric drugs such as furosemide or methylxanthines, both of which increase calcium loss, and steroids, which decrease bone formation by supporting osteoclast differentiation and inhibiting osteoblast growth. No patient in our sample received theophylline and virtually all received caffeine. Similarly, only 12 patients received a diuretic drug and only 4 received furosemide, and therefore it was not possible to explore differences in the incidence of MBD in the context of treatment with these drugs. The use of postnatal steroids for BPD was uncommon (21 patients, 9.6%) and we observed a non-significant trend towards increased use of steroids in the MBD group (19% vs. 8.4%). Moreover, postnatal steroid treatment usually began after biochemical screening for MBD in our cohort (median, 26.5 and 19.7 days, respectively), hindering proper examination of this association.

Contrary to some previous reports [2], we observed no significant association between the incidence of MBD and BPD, although the unadjusted analysis revealed certain trends and some data from patients that met the high-risk criteria that are worth mentioning. Although non-significant, the incidence of BPD in MBD infants was almost double that of non-MBD infants (30% vs. 17%) and the duration of supplementary oxygen was longer, at the limit of significance (408 vs. 120 h, *p* = 0.052). High-risk infants also had worse respiratory outcomes in terms of rate of surfactant administration (47% vs. 33%, *p* = 0.04), rate of mechanical ventilation (34% vs. 21%, *p* = 0.04), incidence of BPD (28% vs. 11%, *p* = 0.003), duration of non-invasive ventilation (144 vs. 96 h, *p* = 0.009), and duration of supplementary oxygen (384 vs. 72 h, *p*-value < 0.001).

Common neonatal morbidities can be either a cause or consequence of MBD, and it can be difficult to differentiate their exact role in the underlying pathophysiology. Do our findings simply reflect the different GA between groups? Do they reflect a worse respiratory outcome among patients with MBD or merely a higher incidence of MBD among patients with BPD? Our study design and sample size make these questions difficult to answer. Nonetheless, the findings are interesting and can be used to generate new hypotheses. One mechanism that could account for the association between MBD and chronic lung disease is inadequate mineral intake together with fluid restriction and more prolonged use of diuretics or systemic steroids, in addition to muscle weakness due to hypophosphatemia.

Some limitations of our study should be noted. Given its partially retrospective design we do not have reliable data on nutrient intake during the first days of life that could potentially influence the results of the biochemical screening for MBD. Data on iron metabolism and RBC transfusion practices are similarly lacking. The absence of a bone imaging method to diagnose MBD is another limitation. In our opinion, standard radiographs are of limited utility for preventive purposes, as they reveal alterations only after a major reduction in bone mineralization [14,15]. Furthermore, identifying osteopenia radiologically is difficult, as there is considerable interobserver variability and clinical interpretation can be imprecise [10]. DXA, which is whole body for preterm infants, and quantitative ultrasound are performed primarily for research purposes and were not available during the study period in the units that participated in our study. Hence, biochemical criteria based on serum ALP and P were used as surrogates to diagnose MBD. As shown in recent surveys, these parameters are the most commonly used in screening tests [6,14,16], and their combination has shown good sensitivity for the diagnosis of MBD [19,34,46]. However, we must acknowledge that our criteria may have led to overestimation of the true incidence of MBD in our cohort.

## 5. Conclusions

In conclusion, despite current nutrition practices, the incidence of MBD in very preterm infants remains notable, affecting more than 1 in 10 infants according to early biochemical criteria (serum ALP and phosphate). This study provides evidence of contemporaneous risk factors for MBD of prematurity, and concludes that birth weight is the parameter most strongly associated with MBD.

More research is needed to further our understanding of MBD, to detect newborns who are at high risk and require specific screening and therapeutic interventions, and to determine the precise role of RBC transfusions in the pathophysiology of MBD.

## Figures and Tables

**Table 1:** Characteristics of the study population.

Demographic and Perinatal Variables		Respiratory Support	
**Gestational age, week**	29.9 (1.9) (28.8–31.4)	Intubation in delivery room	36 (16.5)
**Female**	125 (57.3)	Surfactant	85 (39)
**Prenatal steroids (complete)**	183 (83.9)	NIV during admission	209 (95.9)
**Chorioamnionitis**	46 (21.1)	MV during admission	59 (27)
**Maternal arterial hypertension**	52 (23.9)	Duration of MV, h (n = 59)	144.1 (213.1) (19–144)
**Multiple birth**	83 (38.1)	Duration of NIV, h (n = 209)	176.3 (188) (48–216)
**IVF**	65 (29.8)	Duration of supplementary oxygen (hours)	461.9 (661.5) (24–708)
**Caesarean section**	169 (77.5)	**Clinical outcomes**	
**Apgar 1 min**	6.8 (1.8) (6–8)	BPD	41 (18.8)
**Apgar 5 min**	8.2 (1.3) (8–9)	BPD moderate-severe	18 (8.3)
		Medically treated PDA	25 (11.5)
**Nutrition and growth**		Surgically treated PDA	12 (5.5)
**Birth weight, g**	1207.5 (245.3) (1011–1430)	GM-IVH (all grades)	30 (13.8)
**Birth weight, z-score**	−0.47 (0.77) (−0.99–0.06)	IVH ≥ Grade 3	6 (2.8)
**Small for gestational age**	15 (6.9)	Late-onset sepsis	62 (28.4)
**Weight at discharge, g**	2437.2 (365.3) (2230–2588)	NEC	7 (3.2)
**Weight at discharge, z-score**	−1.3 (1.0) (−1.8–−0.7)	Surgical NEC	4 (1.8)
**Weight gain, g/kg/day**	14.5 (21.5) (11.4–14.8)	Leukomalacia	15 (6.9)
**Postnatal growth restriction**	89 (40.8)	ROP	43 (14.6)
**Parenteral nutrition**	218 (100)	ROP grade III-IV	5 (2.4)
**Days on parenteral nutrition**	10.4 (6.8) (6–12)	Intensive care stay, d	24.0 (18.7) (10–34)
**Start of enteral feeding, days of age**	1.9 (1.3) (1–2)	Hospital stay, d	57.6 (53.1) (40–66)
**Type of enteral feeding**		Red blood cell transfusion	96 (44)
**Breast milk**	117 (53.7)		
**Donor breast milk**	47 (21.6)		
**Formula**	54 (24.7)		
**Breast milk fortification**	186 (85.3)	**Biochemical MBD screening**	
**Start of fortification, days of age (n = 186)**	12.2 (6.3) (9–14)	Age at biochemical screening, days	19.7 (7.1) (15–24)
		Serum creatinine, mg/dL	0.42 (0.19) (0.27–0.56)
**Medications**		Serum phosphorus, mg/dL	6.2 (1.0) (5.7–7)
**Caffeine**	214 (98.2)	214 (98.2)	9.8 (0.5) (9.5–10.2)
**Inotropes**	16 (7.3)	Serum magnesium, mg/dL (n = 166)	2.1 (0.5) (1.9–2.2)
**Steroids for BPD**	21 (9.6)	Serum ALP, IU/L	832 (354) (582–1019)
**Start of steroids for BPD, days of age (n = 21)**	26.6 (16.5) (17–32)	PTH, pg/mL (n = 143)	89.1 (67.7) (44–121)
**Furosemide**	4 (1.8)	Serum ALP > 900 IU/L	77 (35.3)
**Thiazides**	9 (4.1)	Serum phosphorus > 5.5 mg/dL	49 (22)
**Spironolactone**	3 (1.4)	MBD	27 (12.3)
**Days on diuretics (n = 12)**	11.0 (18.5) (5–35)	High screening risk for MBD	96 (44)
**Oral phosphorus supplements**	44 (20.2)	Hyperparathyroidism	59 (27.1)
**Oral calcium supplements**	124 (56.9)		
**Oral vitamin D supplements**	218 (100)		

All values are expressed as n (%) for qualitative variables and as the mean (SD) (IQR) for quantitative variables. IVF, in vitro fertilization; MV, mechanical ventilation; NIV, non-invasive mechanical ventilation; NEC, necrotizing enterocolitis; BPD, bronchopulmonary dysplasia; PDA, patent ductus arteriosus; GM-IVH, germinal matrix-intraventricular hemorrhage; ROP, retinopathy of prematurity; ALP, alkaline phosphatase; PTH, parathyroid hormone; MBD, metabolic bone disease.

**Table 2:** Univariate analyses comparing infants with metabolic bone disease (cases) versus controls, and infants with a high versus low risk for metabolic bone disease.

	MBD			High-Risk for MBD		
	No N = 191 ^1^	Yes N = 27 ^1^	*p*-Value ^2^	No N = 122 ^1^	Yes N = 96 ^1^	*p*-Value ^2^
**Demographic and perinatal variables**						
**Gestational age, week**	30.57 (28.86–31.57)	29.14 (27.5–30.14)	**0.005**	30.86 (29.28–31.71)	29.57 (28.0–30.79)	**<0.001**
**Female**	113 (59%)	12 (44%)	0.2	74 (61%)	51 (53%)	0.3
**Prenatal steroids (complete)**	159 (83%)	24 (89%)	0.3	100 (82%)	83 (86%)	0.6
**Chorioamnionitis**	42 (22%)	4 (15%)	0.5	27 (22%)	19 (20%)	0.8
**Maternal hypertension**	44 (23%)	8 (30%)	0.6	26 (21%)	26 (27%)	0.4
**Multiple birth**	75 (39%)	8 (30%)	0.5	48 (39%)	35 (36%)	0.8
**IVF**	58 (30%)	7 (26%)	0.8	35 (29%)	30 (31%)	0.8
**Caesarean section**	147 (77%)	22 (81%)	0.8	93 (76%)	76 (79%)	0.7
**Apgar 1 min**	7 (6.00, 8.00)	7 (5.00, 7.00)	0.11	7 (6.00, 9.00)	7 (6.00, 8.00)	**0.042**
**Apgar 5 min**	8 (8.00, 9.00)	8 (8.00, 8.00)	0.095	8 (8.00, 9.00)	8 (8.00, 9.00)	0.064
**Nutrition and growth**						
**Birth weight, g**	1280 (1099–1440)	921 (850–1230)	**<0.001**	1330 (1150–1460)	1120 (908–1300)	**<0.001**
**Birth weight, z-score**	−0.46 (−0.96, 0.09)	−0.87 (−1.27, −0.49)	**0.024**	−0.43 (−0.92, 0.03)	−0.59 (−1.05, 0.08)	0.4
**Small for gestational age**	13 (6.8%)	2 (7.4%)	0.9	6 (4.9%)	9 (9.4%)	0.3
**Maternal Body Mass Index**	25.57	27.27	0.2	25.59	26.04	0.6
**Weight at discharge, kg**	2.37 (2.24–2.59)	2.32 (2.2–2.54)	0.6	2.36 (2.24–2.54)	2.36 (2.20–2.67)	0.7
**Weight gain, g/kg/day**	14.07 (11.34, 14.62)	14.56 (12.75, 15.75)	0.8	13.35 (11.51, 14.84)	13.06 (11.25, 14.83)	0.7
**Postnatal growth restriction**	81 (42%)	8 (30%)	0.3	42 (34%)	47 (49%)	**0.043**
**Days on PN**	8 (6, 11)	10 (7, 20)	**0.045**	7 (6, 10)	10 (7, 15)	**<0.001**
**Start of eteral feeding start, days of age**	2.00 (1–2)	200 (1–2)	0.8	2.00 (1–2)	2.00 (1–2)	0.4
**Breast milk**	141 (74%)	23 (85%)	0.3	90 (74%)	74 (77%)	0.7
**Breast milk fortification**	161 (84%)	25 (93%)	0.4	103 (84%)	83 (86%)	0.8
**Respiratory support**						
**Intubation in delivery room**	29 (15%)	7 (26%)	0.2	17 (14%)	19 (20%)	0.3
**Surfactant**	73 (38%)	12 (44%)	0.7	40 (33%)	45 (47%)	**0.048 (0.14)**
**NIV during admission**	182 (95.3%)	27 (100%)	0.4	115 (94.3%)	94 (97.9%)	0.3
**MV during admission**	50 (26%)	9 (33%)	0.6	26 (21%)	33 (34%)	**0.045 (0.15)**
**Duration of MV, h (n = 59)**	48 (18, 120)	24 (20, 324)	0.9	27 (18, 85)	72 (20, 324)	0.3
**Duration of NIV, h (n = 209)**	96 (51, 186)	120 (60, 402)	0.3	96 (48, 144)	144 (72, 330)	**0.009**
**Duration of supplementary oxygen, h (n = 135)**	120 (19, 654)	408 (96, 888)	0.052	72 (12, 216)	384 (60, 1074)	**<0.001**
**Medications**						
**Caffeine**	187 (98%)	27 (100%)	>0.9	118 (97%)	96 (100%)	0.13
**Inotropes**	12 (6.3%)	4 (15%)	0.12	4 (3.3%)	12 (12%)	**0.02 (0.18)**
**Steroids for BPD**	16 (8.4%)	5 (19%)	0.2	7 (5.7%)	14 (15%)	**0.049 (0.15)**
**Start of steroids for BPD, days of age (n = 21)**	22 (16, 30)	31 (23, 33)	0.2	21 (16, 23)	30 (21, 33)	0.14
**Clinical outcomes**						
**BPD**	33 (17%)	8 (30%)	0.2	14 (11%)	27 (28%)	**0.003 (0.21)**
**BPD moderate-severe**	15 (7.9%)	3 (11%)	0.5	7 (5.7%)	11 (11%)	0.2
**Medically treated PDA**	171 (90%)	22 (81%)	0.2	115 (94%)	78 (81%)	**0.005 (0.2)**
**IVH ≥ Grade 3**	4 (2.1%)	2 (7.4%)	0.2	2 (1.6%)	4 (4.2%)	0.4
**Late-onset sepsis**	49 (26%)	13 (48%)	**0.028** **(0.16)**	26 (21%)	36 (38%)	**0.013 (0.18)**
**NEC**	5 (2.6%)	2 (7.4%)	0.2	3 (2.5%)	4 (4.2%)	0.7
**Leukomalacia**	10 (5.2%)	5 (19%)	**0.025** **(0.17)**	9 (7.4%)	6 (6.2%)	>0.9
**ROP**	14 (7.8%)	2 (7.7%)	>0.9	5 (4.4%)	11 (12%)	0.075
**NICU stay, days**	20 (10, 34)	18 (10, 31)	0.9	20 (10, 32)	20 (10, 34)	0.5
**Hospital stay, days**	48 (40, 64)	64 (47, 76)	**0.012**	44 (36, 58)	58 (46, 74)	**<0.001**
**Red blood cell transfusion**	76 (40%)	20 (74%)	**0.002** **(0.23)**	35 (29%)	61 (64%)	**<0.001 (0.35)**
**Biochemical MBD screening**						
**Age at biochemical screening, days**	16 (15, 25)	16 (15, 17)	0.5	16 (15, 30)	16 (15, 17)	0.08
**Serum Ca, mg/dL**	9.90 (9.60, 10.28)	9.60 (9.30, 9.95)	**0.002**	10.00 (9.70, 10.3)	9.70 (9.30, 10.03)	**<0.001**
**Serum Mg, mg/dL**	2.08 (1.94, 2.20)	2.02 (1.90, 2.21)	0.9	2.06 (1.95, 2.16)	2.09 (1.91, 2.23)	0.5
**PTH, pg/mL (n = 143)**	74 (47, 123)	56 (34, 96)	0.11	64 (47, 123)	77 (40, 110)	0.9
**25-OH-D, ng/mL**	29 (21, 37)	28 (22, 40)	0.6	28 (20, 38)	29 (24, 37)	0.2
**Hyperparathyroidism**	53 (28%)	6 (22%)	0.7	30 (25%)	29 (30%)	0.4

^1^ All values are expressed as n (%) for qualitative variables and as the mean (SD) (IQR) for quantitative variables. ^2^ Statistical tests performed: Wilcoxon rank-sum test; Student’s *t*-test; Yuen test for trimmed means; chi-squared test of independence; Fisher’s exact test. Cramer’s V values are presented after *p*-values where appropriate. Bold in p-values indicates statistical significance. Ca, calcium; IVF, in vitro fertilization; PN, parenteral nutrition; MV, mechanical ventilation; NIV, non-invasive mechanical ventilation; NEC, necrotizing enterocolitis; BPD, bronchopulmonary dysplasia; PDA, patent ductus arteriosus; P, phosphorus; PTH, parathyroid hormone; Mg, magnesium; Ca, calcium; 25-OH-D, serum 25-OH-vitamin D; GM-IVH, germinal matrix-intraventricular hemorrhage; ROP, retinopathy of prematurity; MBD, metabolic bone disease.

## Supplementary Materials

The following are available online at https://www.mdpi.com/2072-6643/12/12/3786/s1: Table S1. Urinary excretion of calcium and phosphate in spot urine samples.
