# Supplementary material for: Metabolic Bone Disease of Prematurity: Risk Factors and Associated Short-Term Outcomes
Source: Nutrients. 2020 Dec 10;12(12):3786. doi: 10.3390/nu12123786 (PMC7764323; doi:10.3390/nu12123786)
Supplement: Supplementary file 1 [file nutrients-12-03786-s001.pdf]

Table S1. Urinary excretion of calcium and phosphate in spot urine samples (n=71).

	MBD			*High risk for MBD*		
	No, N = 64	*Yes, N = 7*	*p-value*	*No, N = 39*	*Yes, N = 32*	*p-value*
**Age at screening, days**	15 (15, 25)	16 (15, 17)	0.5	16 (15, 30)	16 (15, 17)	0.08
**Serum creatinine, mg/dl**	0.38 (0.27, 0.54)	0.45 (0.32, 0.64)	0.3	0.37 (0.24, 0.50)	0.42 (0.31, 0.63)	**0.017**
**Serum calcium, mg/dl**	9.90 (9.60, 10.28)	9.60 (9.30, 9.95)	**0.002**	10.0 (9.70, 10.30)	9.70 (9.30, 10.03)	**<0.001**
**Serum P, mg/dl**	6.50 (5.95, 7.00)	5.00 (4.05, 5.20)	**<0.001**	6.80 (6.40, 7.20)	5.65 (5.00, 6.23)	**<0.001**
**Serum ALP (IU/L)**	735 (556, 911)	1211 (1064, 1484)	**<0.001**	624 (502, 755)	1050 (927, 1269)	**<0.001**
**Urinary P, mg/dl**	8 (4, 16)	3 (0, 6)	**0.037**	6 (4, 14)	9 (4, 16)	0.7
**Urinary calcium, mg/dl**	5.6 (4.2, 8.4)	3.9 (3.0, 11.4)	0.6	5.2 (4.0, 7.8)	6.9 (3.9, 10.0)	0.2
**Urinary creatinine, mg/dl**	10 (7, 14)	14 (9, 14)	0.3	9 (7, 13)	10 (8, 15)	0.6
**Urinary calcium/creatinine**	0.58 (0.40, 0.89)	0.39 (0.21, 0.95)	0.5	0.51 (0.40, 0.67)	0.72 (0.38, 1.09)	0.068
**Urinary P/creatinine**	0.84 (0.35, 1.74)	0.08 (0.03, 0.59)	**0.014**	0.71 (0.35, 1.74)	0.98 (0.29, 1.57)	>0.9
**Urinary P/calcium, mg/mg**	1.54 (0.55, 3.54)	0.77 (0.07, 1.93)	0.2	1.25 (0.60, 3.79)	1.73 (0.48, 3.08)	0.6
**Phosphorus tubular reabsorption (%)**	94.7 (90.3, 97.8)	98.5 (96.4, 99.8)	0.066	95.8 (92.5, 97.9)	94.1 (87.9, 97.7)	0.3

[1]All values are expressed as the mean (IQR). [2]Statistical tests performed: Wilcoxon rank-sum test, Student's t-Test, Yuen test for trimmed means. P, phosphate; ALP, alkaline phosphatase. .
